# Design and Evaluation of a Real-Time Audio Source Separation Algorithm to Remix Music for Cochlear Implant Users

**DOI:** 10.3389/fnins.2020.00434

**Published:** 2020-05-19

**Authors:** Sina Tahmasebi, Tom Gajȩcki, Waldo Nogueira

**Affiliations:** Department of Otolaryngology, Medical University Hannover and Cluster of Excellence “Hearing4all”, Hanover, Germany

**Keywords:** music source separation, deep learning, neural networks, multi-layer perception, real-time, cochlear implant

## Abstract

A cochlear implant (CI) is a surgically implanted electronic device that partially restores hearing to people suffering from profound hearing loss. Although CI users, in general, obtain a very good reception of continuous speech in the absence of background noise, they face severe limitations in the context of music perception and appreciation. The main reasons for these limitations are related to channel interactions created by the broad spread of electrical fields in the cochlea and to the low number of electrodes that stimulate it. Moreover, CIs have severe limitations when it comes to transmitting the temporal fine structure of acoustic signals, and hence, these devices elicit poor pitch and timber perception. For these reasons, several signal processing algorithms have been proposed to make music more accessible for CI users, trying to reduce the complexity of music signals or remixing them to enhance certain components, such as the lead singing voice. In this work, a deep neural network that performs real-time audio source separation to remix music for CI users is presented. The implementation is based on multi-layer perception (MLP) and has been evaluated using objective instrumental measurements to ensure clean source estimation. Furthermore, experiments in 10 normal hearing (NH) and 13 CI users to investigate how the vocals to instruments ratio (VIR) set by the tested listeners were affected in realistic environments with and without visual information. The objective instrumental results fulfill the benchmark reported in previous studies by introducing distortions that are shown to not be perceived by CI users. Moreover, the implemented model was optimized to perform real-time source separation. The experimental results show that CI users prefer vocals 8 dB enhanced with the respect to the instruments independent of acoustic sound scenarios and visual information. In contrast, NH listeners did not prefer a VIR different than zero dB.

## 1. Introduction

A cochlear implant (CI) is a medical electronic device that is surgically implanted in the inner ear and can provide hearing sensations to people suffering from profound hearing loss. CIs allow the patients to understand speech in quiet and even in a noisy background. However, music appreciation is still challenging for CI users, as it requires a good pitch perception and melody recognition (McDermott, 2004; Limb and Roy, 2013). CI devices typically transmit 12–22 spectral channels, each modulated slowly in time. This representation provides enough information for speech understanding in quiet conditions and rhythmic perception of music. However, this representation is not enough to support speech understanding in noise or melody recognition, as melody recognition requires complex pitch perception, which in turn depends strongly on access to spectral and temporal fine structure cues (McDermott, 2004; Macherey et al., 2011). This work investigates the use of a real-time algorithm to make music more accessible for CI users.

Previous research in the area of music enhancement for CI users has focused on reducing music complexity (Nagathil et al., 2017) or on amplifying vocals relative to the background instruments (Buyens et al., 2014; Pons et al., 2016; Gajȩcki and Nogueira, 2018). Spectral complexity reduction of music was investigated based on dimensionality reduction techniques, such as principal component analysis and a partial-least squares analysis. Enhancement of singing voice has been investigated based on the finding that CI users prefer singing music remixed such that the vocals are boosted by 6 dB with respect to the background instruments (Buyens et al., 2014). In this context several source separation algorithms have been proposed to separate the vocals from the instruments and remix these components accordingly. Previous approaches used a harmonic/percussive sound separation (HPSS; Buyens et al., 2014) algorithm, non-negative matrix factorization (NMF), multi-layer perceptrons (MLP), deep recurrent neural networks (DRNN), and convolutional autoencoders (DCAE) in order to separate different sources within an audio mixture (Pons et al., 2016; Gajȩcki and Nogueira, 2018). However, all these algorithms were implemented in non-real-time fashion to perform source separation.

A key factor in the design of source separation methods for music enhancement is the distortions that these algorithms introduce in the processed signals. These distortions are typically quantified through objective instrumental measures, such as the signal-to-distortion ratio (SDR), the signal-to-artifacts ratio (SAR), and the signal-to-interference ratio (SIR) (Vincent et al., 2010). Gajȩcki and Nogueira (2018) investigated the maximum levels of artifacts and distortions accepted by CI users to remix music with enhanced vocals. They demonstrated that source separation algorithms with an SDR > 0.69 dB and an SAR > 4.42 dB were suitable for remixing singing pop music for CI users.

In order for the source separation algorithm to operate in real-time, algorithmic complexity and latency need to be minimized. Regarding latency, even delays in the order of tens of milliseconds can cause a de-synchronization between the visual and the acoustic information provided by the CI. Stone and Moore (2003) measured maximum non-noticeable latency for hearing aid devices of around 15–20 ms for speech signals. The international telecommunication union (ITU) performed several subjective evaluations (ITU, 2008) and reported the acceptable and the detectable lip synchronization error. This error was assessed by means of the time delay between the visual feedback and acoustic information provided by a person speaking. Their results revealed that the measured time delay detectability was 125 ms and the threshold of acceptability was 185 ms, respectively, with respect to perfect lip synchronization when audio lagging behind the video. Furthermore, Hay-McCutcheon et al. (2009), in a similar study, investigated the audiovisual asynchrony for CI users. In this study, the measured minimum noticeable audiovisual asynchrony was around 200 ms, when the video was leading the audio. These values could be taken as an upper boundary for the design of a real time source separation algorithm.

Previous source separation algorithms to remix music for CIs have been evaluated under laboratory settings using clean digital recordings. It remains a question whether these algorithms are usable in real music events in which reverberation influences the acoustics. Moreover, these source separation algorithms were evaluated only using pre-processed sounds. It is therefore interesting to investigate whether these algorithms are usable in real-time giving the users the possibility to continuously modify the level difference between the singing voice and the instruments to reach a final decision on their preference.

Although music experiments are typically investigated only through sound, it is very important to consider that CI users have access to both hearing and visual cues. It has been shown that when congruent visual and auditory cues are processed together, perceptual accuracy is enhanced in both normal hearing (NH) and in hearing-impaired listeners (Perrott et al., 1990; Ross et al., 2007; Landry et al., 2012). The importance of vision in lip reading the singing voice, which helps in understanding the lyrics, the fact that CI users can see the instruments being played, as well as the information provided by the performance can significantly enhance their perception (Plant, 2015). It therefore remains a question of whether source separation algorithms used to enhance the vocals are necessary if visual information is available.

In this work, we implement and evaluate a deep neural network (DNN) to perform real-time music source separation to improve music appreciation of CI users in realistic acoustic scenarios. The model is based on an MLP and is trained to automatically identify the lead singing voice contained in western pop music to remix it accordingly to the subjects' preference. Prior to assessing individual balance preferences, objective instrumental measures will be used to make sure that the source separation algorithm fulfills the benchmark proposed by Gajȩcki and Nogueira (2018). Finally, the balance between music and singing voice will be assessed by means of experimental tests were subjects will indicate their preferred vocals-to-instruments-ratio (VIR), which is defined as the ratio between the power of the vocal signal and the instruments signal in dB, with and without visual cues. As the experiments were meant to be as realistic as possible, 360° video and 3D audio were provided to the listeners. In this context, the subjects should be able to move their heads toward the singer or to the background instruments and therefore they should be provided with consistent acoustic and visual cues.

## 2. Methods and Materials

### 2.1. Music Source Separation and Remix Algorithm

**Figure 1:**
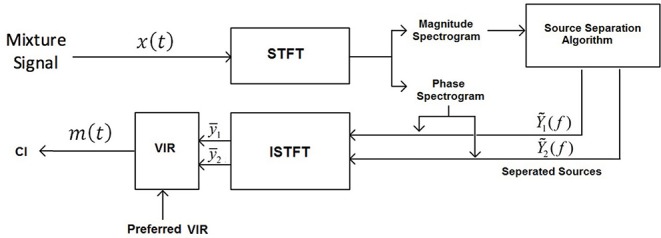
Block diagram of the source separation algorithm.

Figure 1 shows a block diagram representing the data-flow of the used music source separation and remixing framework. The input signal *x*(*t*) consists of the original mixture, of vocals and instruments and the output signal *m*(*t*) is a remixed version of the input signal that is delivered to the CI speech processor at discrete time *t*. The desired VIR is applied to the estimated signals to be then remixed and delivered to the CI listener.

The music source separation process starts by feeding the magnitude spectrogram of the input mixture to the source separation algorithm. For a vocal signal *y*_1_(*t*) and an instruments signal *y*_2_(*t*) we construct a corresponding mixture signal *x*(*t*) = *y*_1_(*t*)+*y*_2_(*t*). We compute the short-time Fourier transform of length 1024 samples to obtain the spectrums *Y*_1_(*k*), *Y*_2_(*k*), *X*(*k*) for frame *k*. Since the time-domain audio signals are real, we used half of the spectral length, as dropping the negative frequencies does not lead to any information loss, which leads to a spectral size of 513 bins. For inverting the spectrum of *Y*_1_(*k*) and *Y*_2_(*k*), back into the time domain, we used the phase of the mixture spectrum and applied the inverse STFT with overlap-add to synthesize the music signal, for both the vocals and instruments components. Figure 1 illustrates the block diagram of the algorithm. The ability that MLPs have to approximate any input/output map made them one of the most popular network architectures (Panchal et al., 2011). Furthermore, as MLPs require relatively low computation complexity, they became the first choice of this study to perform music source separation. The number of input and output units is directly related to the size of the analysis window. We used an STFT with a window length of 1,024 samples, an overlap of 75%, and a Hamming window to transform, which leads to a spectrum with a dimension of 513 bins. In order to exploit the temporal dependency in the music signals, three consecutive frames with two frames overlap were used as input to the network resulting in an input size of 513*3. During the source separation process, we specify multiple parameters, that have a direct effect on separation quality and are linked to the network's structure. The depth of the MLP was set to one hidden layer with 1,024-units and a rectified linear unit (ReLU) activation function, which resulted in a three layer network with an input size of 513*3 units and output layer of 1,026 (2*513) units which corresponds to the two spectrums of vocal and instruments signal. After selecting the number of hidden layers and units in each layer, a proper training algorithm was used to minimize the algorithm error by fitting the model to the training data. During training, the fixed parameters were: the batch size which was set to 128 and the initial learning rate which was set to 0.005. The adaptive moment estimation algorithm (Adam) (Kingma and Ba, 2014) was used as the optimization algorithm. A hundred epochs were used to train the network, where after each epoch, the learning rate was decreased by 0.9 and the training data were shuffled. To avoid over-fitting, a dropout layer with a probability of 80% was applied. Three consecutive frames were used as input to exploit the temporal context in the audio signals. However, introducing more frames to the network did not improve the objective instrumental measurement values.

### 2.2. Audio Material Used to Train the Neural Network

In this work, we use three audio data sets to perform the objective and experimental evaluations in NH and CI users of the investigated audio source separation algorithms. The data sets will be described in the following lines.

**Figure 2:**
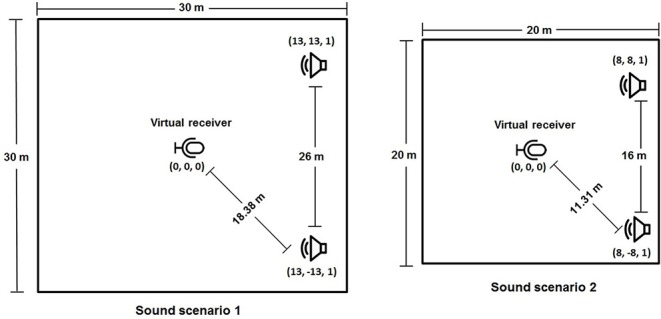
Visualization of the created sounds scenarios. **(Left)** Sound scenario 1 consisting of large reverberant room (T20 = 0.24 s, T60 = 0.65 s); **(Right)** Sound scenario 2 consisting of a smaller and less reverberant room (T20 = 0.18 s, T60 = 0.5 s).

- **iKala Data Set:** The first data set introduced by Chan et al. (2015) namely iKala contains 252 30-s tracks of vocal and backing track music with a sample rate of 44,100 Hz. Each music track is a stereo recording, with one channel for the singing voice and the other for background music. All music tracks have been performed by professional musicians and six singers, of which three were female and three males. The iKala data set contains non-vocal time passages where the source separation algorithm assumes the presence of vocals and having non-vocal time passages in the data set may challenge the algorithm. The presence of vocals in the instruments signal and long non-vocals regions were the reasons that in experimental settings 30 music tracks of data set have been excluded, making the total number of music tracks in the iKala data set equal to 222.
- **The MUSDB Data Set:** MUSDB data set was the second data set with 150 professionally mixed songs from different genres, each including four stereo sources (bass, drums, vocals and a group of other instruments) used in this work. The data set was divided into the training and the testing data set with 100 and 50 songs, respectively.
- **Buyens Shared Data Set:** As the third data set, six popular music pieces (Buyens et al., 2014) that have been used in previous CI studies to create and report a benchmark (Pons et al., 2016; Gajȩcki and Nogueira, 2018) have been also used in this study.
- **Custom Data Set with Virtual Acoustics:** All the previous data sets were studio recordings with no spatial characteristics. In order to have a DNN which can be used in a realistic sound scenario, it was necessary to train the model with a data set that included spatial characteristics. To create a music data set with such characteristics, TASCAR was used to simulate realistic sound scenarios (Grimm et al., 2015). TASCAR is a toolbox to create virtual acoustics in real-time. TASCAR toolbox is based on an image source model that simulated localized sources, reflections and a diffuse sound model for adding background recordings and reverberation. The image source model rendered an image source for each combination of the primary sound source and a reflecting surface, provided that the primary source was not behind the surface. To playback the content of a virtual scene created by TASCAR on an arbitrary playback device, Ambisonics decoding must be performed to get the signals for the channels of the playback system. The acoustic scene was rendered and played through an array of 16 loudspeakers. In order to assess the effect of different sound scenarios on the performance of the DNN model, two rooms with different dimensions and acoustic characteristics were rendered through the 16 loudspeaker setup and the TASCAR toolbox. A reverberant room with a T20 = 0.24 s and T60 = 0.65 s and a smaller and less reverberant room with a T20 = 0.18 s and T60 = 0.5 s were used in the experiments.
  In each sound scenario a virtual receiver with an omnidirectional characteristic is defined which captures the sound in that space. The real receiver (CI user or dummy head) was placed in the center of the loudspeaker layout in the lab and received the sound corresponding with the position of the virtual receiver in each defined sound scenario. The virtual environment consisted of two loudspeakers reproducing stereo mixes aiming at resembling a music concert amplified with a public address system (PA system). The room impulse response was modeled by the toolbox using the reflection coefficient and a damping factor shown in Table 1. Figure 2 shows a visualization of the created sound scenarios.
  A Nucleus speech processor (Cochlear, Sydney, Australia), mounted on a dummy head at the height of 1.4 m was placed in the center of loudspeaker layout to record the custom data set. While TASCAR was running on a Linux operating system, another PC, which was connected to the Nucleus speech processor through a sound card, was recording the simulated sound field. The custom data set was exclusively used to train, validate and, test the MLP using objective instrumental measures.

**Table 1:** Details about created sound scenarios.

**Sound scenario**	**Dimensions in meters**	**Damping factor**	**Reflection coefficient**	**Virtual receiver position**
1	(30, 30, 8)	0.70	0.25	At the center (0, 0, 0)
2	(20, 20, 7)	0.80	0.20	At the center (0, 0, 0)

#### 2.2.1. Training, Validation, and Testing Data Set

To have a uniform data set, the signals contained in the MUSDB set, which had different lengths, were chopped in 30 s long samples and were mixed with the iKala data set. Finally, we distributed the iKala and MUSDB data sets into the training, validation and testing sets. During this study, 60% of the iKala and MUSDB data sets were used as the training data set, 20% as the validation data set and 20% as testing data set. Moreover, the music signals shared by Buynes were added to the testing data set.

### 2.3. Experiments in Normal Hearing Listeners and Cochlear Implant Users

This study consisted of two experiments. In the first one, the effect of having different sound scenarios on VIR preferences was investigated. In the second one, the effect of having visual cues on VIR preferences was investigated.

#### 2.3.1. Subjects

Thirteen bilateral CI users with different musical backgrounds and 10 NH subjects participated in the study. The demographic information of the tested CI subjects is presented in Table 2. From the 13 CI users only 10 CI subjects participated in each experiment as indicated in the same table. All subjects gave informed consent to the project as approved by the Medical University Hannover Institutional Review Board. The subjects were asked to turn off any program on the speech processor and use the audio cable as input. None of the subjects had residual hearing except for Subject S07, who had bilateral residual hearing up to 250 Hz. This particular subject was also asked to wear soft foam earplugs to minimize acoustic leakage. In the first perceptual experiment, ten NH subjects participated in the study as a control group.

**Table 2:** Information of the CI subjects, who took part in the experiments.

**Subject id**	**Age**	**Cause of deafness**	**Duration of deafness**	**Implant experience on tested side**	**Sound processor**	**Brand**
S01^a, b^	65–70	Unknown	9Y	21Y	Opus2	MEDEL
S02^a, b^	80–85	Genetic	4M	7Y	Opus2	MEDEL
S03^a, b^	55–60	Sudden deafness	2M	4Y	Naida	AB
S04^a, b^	60–65	Unknown	6Y	13M	CP910	Cochlear
S05^a, b^	70–75	Sudden deafness	29M	3Y	CP910	Cochlear
S06^a^	65–70	Unknown	3.5Y	5Y	Harmony	AB
S07^a^	65–70	Unknown	4M	12Y	CP910	AB
S08^a, b^	20–25	Otitis media	4Y	20Y	CP910	Cochlear
S09^a^	60–65	Unknown	5Y	4Y	CP910	Cochlear
S10^a, b^	20–25	Unknown	0M	18M	Naida	AB
S11^b^	70–75	Unknown	4Y	4Y	CP910	Cochlear
S12^b^	65–70	CRS	31Y	6Y	CP910	Cochlear
S13^b^	20–25	Meningitis	4 M	18Y	CP910	Cochlear

*Time data are expressed in years (Y) or in months (M)*.

a Participated in the first part of experiment 1

b *Participated in the second part of experiment 1. CRS, congenital rubella syndrome*.

#### 2.3.2. Test Setup Used in Listening Experiments

The experiments were conducted monaurally using the better performing side of each subject. Sound material was presented in a double-walled sound-treated room using 16 active (self-amplified) loudspeakers (Genelec 8030B, Helsinki, Finland), which were organized in a circle with a radius of 1.25 m and were driven by an A16 MKII digital to analog converter connected to a PC with Ubuntu Xenial 14.04 operating system. Subjects were seated in the center of the loudspeaker array and the audio material was always presented at a level corresponding to 65 dB SPL at the position of the participant's head.

The microphone of a Nucleus speech processor (Cochlear, Sydney, Australia) was used to capture the presented audio signals during all subjective tests. The microphone had an omnidirectional characteristic and no beamforming was used during the tests. The captured signals were then processed through the algorithm and presented to the subject's speech processor. A preamplifier was used to amplify the captured signal which was routed to the subject's own processor, in direct-in mode, through a 3.5 mm audio cable. During the tests, the microphone on the subject's CI was disabled.

The subjects were asked to adjust a slider that controlled the VIR using the left and right arrow keys on a keyboard while listening to the music. The slider had 24 steps each corresponding to 1 dB on a logarithmic scale from −12 to +12 dB VIR. In order to prevent any bias, initial VIR for each song presentation was randomly chosen and the subjects were kept blinded to the initial VIR. Afterward, the subjects adjusted the VIR to their preferred setting.

For NH subjects, instead of a CI, a headphone (DT 770, Beyerdynamic, Heilbronn, Germany) was used to present the music tracks. The music tracks presented to the NH subjects were captured with the same microphone on the Nucleus speech processor. The headphone was calibrated to present each music piece at 65 dB SPL using only one side, at the self-reported preferred ear.

#### 2.3.3. Music Material Used in Listening Experiments

Music tracks shown in Tables 3, 4 have been used in experiment 1 and 2, respectively. The music excerpts used in experiment 1 had a duration of 5 s, whereas the ones used in experiment 2 were 45 s in duration. The pieces selected for experiment 2 had clear vocals with simple music accompaniment (electronic in “Cassiopeia” and a flute and a tuba in “der König in Thule”) and in that sense were similar to the pieces used in experiment 1.

**Table 3:** Music tracks used in experiment 1.

**Song id**	**Data set**	**Song name**	**Genre**
M1	iKala	21058_chorus	Pop
M2	iKala	31104_verse	Pop
M3	iKala	31118_chorus	Pop
M4	iKala	54236_chorus	Pop
M5	MUSDB	Secretariat—Over The Top	Pop rock
M6	MUSDB	Georgia Wonder—Siren	Folk rock
M7	MUSDB	The Long Wait—Dark Horses	Folk
M8	Popular music	Hey Jude (The Beatles) (excerpt A)	Pop, country music
M9	Popular music	Hey Jude (The Beatles) (excerpt B)	Pop, country music
M10	Popular music	Dock of the Bay (Otis Redding)	Pop, classic soul

**Table 4:** Music tracks used in experiment 2.

Song id	Song name	Genre
CasA	Cassiopeia (excerpt 1)	Electronic
CasB	Cassiopeia (excerpt 2)	Electronic
CasC	Cassiopeia (excerpt 3)	Electronic
KönigA	Der König in Thule (excerpt 1)	Sung poetry
KönigB	Der König in Thule (excerpt 2)	Sung poetry
KönigC	Der König in Thule (excerpt 3)	Sung poetry

##### 2.3.3.1. *Virtual test scenarios in experiment 1*

The TASCAR library was used in experiment 1 to add spatial characteristics to the studio-quality music tracks. In this experiment the two sound scenarios presented in Figure 2 were used to assess the VIR preferences of NH listerners and CI users.

##### 2.3.3.2. *Virtual test scenarios in experiment 2*

Six music excerpts from live recordings in a concert was used in the experiment 2 (Nogueira, 2019). In comparison to the before-mentioned data sets, which were recorded with studio quality and had no spatial characteristics, these music excerpts were spatially recorded using the Eigenmic 32 microphone (Summit, New Jersey, USA). These music tracks already contained the spatial characteristics of a concert hall.

#### 2.3.4. Experiment 1: Online Vocals to Instruments Ratio Adjustment

In the first experiment, 5-s excerpts of ten signals from three data sets were used (Table 3). Each excerpt was calibrated at 65 dB SPL and was played in a loop until the subject adjusted the VIR slider in their favored position. Subjects had no time limits to adjust the VIR for each music track and were allowed to have a break whenever they desired. After the test, subjects were asked to fill in a questionnaire providing their musical background experiences or knowledge and music genre preference. This procedure including the stimulus length was chosen based on previous studies (Pons et al., 2016; Gajȩcki and Nogueira, 2018). This experiment was divided into two parts. In the first part of the experiment, NH listeners and CI users were asked to adjust the VIR for a test data set. In this part, the VIR was modified keeping the level of the instruments fixed while modifying the level of the vocals. In the second part of the experiment, the VIR was altered by modifying the level of the vocals and the instruments in opposite directions to keep the overall presentation level constant at 65 dB SPL. The second part of this experiment was included to exclude potential effects due to variations in loudness.

#### 2.3.5. Experiment 2: Effect of Visual Information on VIR Preferences

The goal of this experiment was to examine whether visual feedback affects the VIR preferences of the CI recipients. This experiment was divided into two parts. The music pieces 4 of a concert were presented to the subjects, once without visual information, and once with visual information through a set of Oculus Rift (Facebook, Irvine, California, USA) virtual reality (VR) headset. Each music excerpt was repeated until the subjects adjusted the VIR at their favored value. As mentioned in section 2.3.3, the music pieces were recorded with an Eigenmic 32 microphone array that has 32 microphones, capable of providing 4-th order Ambisonics. Reaper (Cockos, New York, USA) on an Ubuntu PC was used as a digital workstation to process the audio material. The audio signals were captured by an Eigenmic 32 microphone array and during the tests they were first encoded to 4-th order Ambisonics and then decoded to the layout used in the testing lab. GoPro player (GoPro, San Mateo, California, USA) on a Windows (Microsoft, Redmond, Washington, USA) PC was deployed to present the visual materials of the concert. As the digital workstation and the GoPro player use different synchronization protocols, a third party application, which was able to send and receive OSC and UDP messages, was used to synchronize both audio and video. For this experiment, the VIR was applied in the same way as in the second part of the previous experiment; i. e. by modifying the level of the vocals and the instruments in opposite directions to keep the overall presentation level constant at 65 dB SPL. During the second part of the test, the Nucleus speech processor was fixed on the Oculus Rift VR headset. It is worth mentioning that five subjects wore eyeglasses, which they took off during this experiment.

## 3. Results

### 3.1. Objective Instrumental Measures

**Figure 3:**
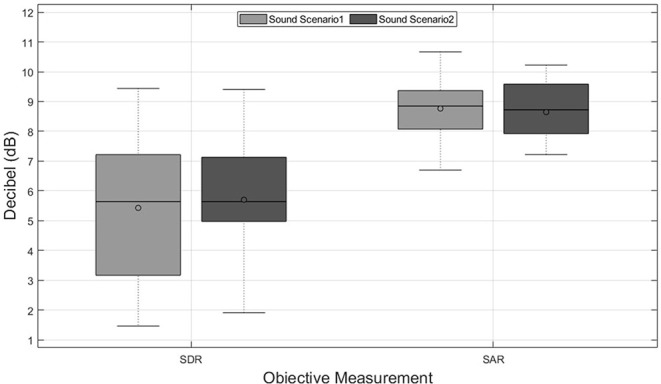
Results of the objective instrumental measurements based on the signal-to-distortion-ratio (SDR) and the signal-to-artifacts-ratio (SAR) in decibels (dB) for two sound scenarios across music tracks.

Figure 3 shows the objective results for music tracks used in the first perceptual experiments. Mean SDR and SAR of the music tracks in sound scenario 1, which was a larger room with more diffusive and reverberant characteristics, with 5.5 dB is around 0.3 dB worse than the mean SDR obtained in the second room. However, the SAR values obtained in the first sound scenario (8.8 dB) were slightly better than in the second sound scenario (8.7 dB). Both sound scenarios fulfill the benchmark reported by Gajȩcki and Nogueira (2018), where the lower bounds for SDR and SAR were reported with 0.69 and 4.42 dB, respectively. It is worth mentioning that the room characteristics cause changes in the music tracks, which leads to different objective results after source separation, which can be seen in Figure 3.

### 3.2. Experiment 1: Online Vocals to Instruments Ratio Adjustment

**Figure 4:**
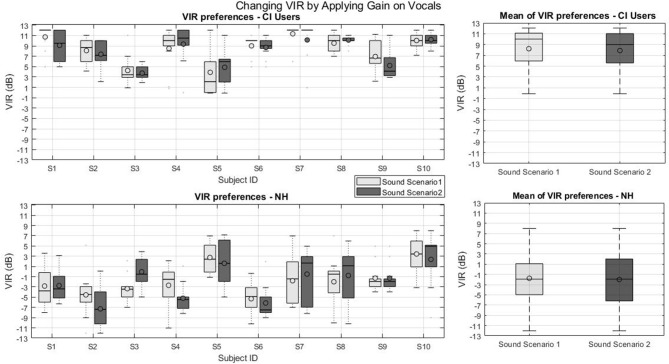
VIR values from part one of experiment 1: Changing VIR by applying gain on vocals. **(Top left)** VIR preferences of CI users, **(Top right)** mean VIR of CI users, **(Bottom left)** VIR preferences of NH subjects, **(Bottom right)** mean VIR of NH subjects.

Figure 4 presents the individual and group VIR preferences of the first part of experiment 1 for CI users (top panels) and NH listeners (bottom panel) for two virtual sound scenarios. The line and the circle in the boxes represent the median and the mean, respectively. On average, the VIR mean across CI users was 8.2 and 8.5 dB and across NH listeners was −2 and −1.7 dB for sound scenarios 1 and 2, respectively. Note that for CI users S1, S6, and S7 no box can be seen in specific sound scenarios as the plots for these subjects collapsed to a single line due to small variance in their results. These subjects adjusted the VIR in more than 75 % of the cases to the same value. R Studio (Boston, Massachusetts, USA) software was used to conduct the statistical analysis. First, the VIR preferences of CI users and NHs were tested against the null hypothesis that the preferred VIR was equal to zero dB. This was done for the two parts of the experiment for each tested sound scenario by means of two-tailed *t*-tests.

For the first part of the experiment, where the VIR was set by modifying the vocals with respect to the instruments, the CI group showed a preference for positive VIRs for the first (*Mean* = 8.5 dB, *SD* = 2.4 dB, *p* < 0.001) and second sound scenarios (*Mean* = 8.2 dB, *SD* = 2.5 dB, *p* < 0.001). The NH group, on the other hand, did not show any evidence of preferring a balance different from the original mix (VIR=0 dB) for the first sound scenario (*Mean* = −2 dB, *SD* = 3.2 dB, *p* < 0.086) nor for the second sound scenario (*Mean* = −1.75 dB, *SD* = 2.8 dB, *p* < 0.082). Finally, none of the tested groups' preferred VIR depended on the sound scenario (*p* = 0.297 for the CI users and *p* = 0.7 for the NH group).

**Figure 5:**
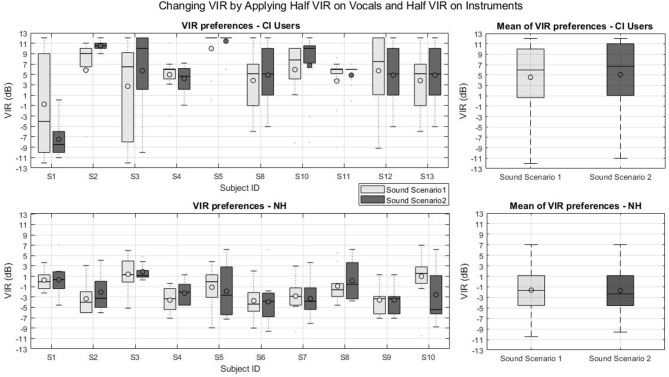
VIR values from part two of experiment 1: Changing VIR by Applying Half VIR on Vocals and Half VIR on Instruments. **(Top left)** VIR preferences of CI users, **(Top right)** mean VIR of CI users, **(Bottom left)** VIR preferences of NH subjects, **(Bottom right)** mean VIR of NH subjects.

Figure 5 presents the individual and group VIR preferences of the second part of experiment 1 for CI users (top panels) and NH listeners (bottom panel) for two virtual sound scenarios. Note that for CI users S1, S6, and S7 no box-plot could be created as they adjusted the VIR in more than 75 % of the cases to the same value. In the second part of experiment 1 subject S5 with around 11 dB and subject S1 with −4 dB had the highest and lowest mean VIR preferences. Subject S1 was the only CI user with a negative mean VIR preferences in all experiments.

For this part of the experiment, where the VIR was set by altering the singing voice and the background instruments level simultaneously in opposite directions. The CI group showed also a preference for positive VIRs for the first (*Mean* = 4.6 dB, *SD* = 2.7 dB, *p* < 0.001) and second sound scenarios (*Mean* = 5.3 dB, *SD* = 5.1 dB, *p* = 0.0093). The NH group, again, did not show any evidence of preferring a balance different from the original mix (VIR = 0 dB) for the first sound scenario (*Mean* = −1.6 dB, *SD* = 2 dB, *p* = 0.0194) nor for the second sound scenario (*Mean* = −1.7 dB, *SD* = 1.9 dB, *p* = 0.0194). For this part of the experiment, again, non of the tested groups' preferred VIR depended on the sound scenario (*p* = 0.024 for the CI users and *p* = 0.027 for the NH group).

To conclude the statistical analysis for this first experiment, a final *t*-test analysis was performed to assess if the measured VIRs depended on the VIR adjustment method (i.e., balancing the vocals alone or adjusting both signals in opposite directions), two-tailed *t*-tests were performed comparing the VIRs measured between the VIR adjustment methods for each group. The *t*-tests revealed that the measured VIRs did not depend on the VIR adjustment method for none of the tested groups *p* < 0.001.

### 3.3. Experiment 2: Effect of Visual Information on VIR Preferences

**Figure 6:**
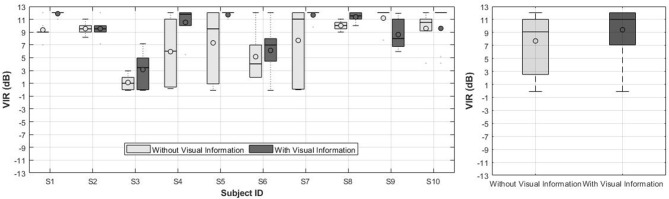
**(Left)** VIR values from 10 CI users in Experiment 2. **(Right)** Mean VIR across subjects and music tracks for Experiment 2.

The individual VIR preferences set by the CI group in the second experiment are presented in Figure 6 (left). Figure 6 (right) shows the mean VIR across subjects and music excerpts for both conditions. The line and the circle in the boxes represent the median and the mean, respectively. As shown in Figure 6, the individual results for the condition without visual information show a larger variance in comparison to the condition with visual information. Similarly to experiment 1 the results from subjects S1, S5, S7, S9, and S10 in some specific sound scenarios are collapsed into a line due to very small variance in their responses.

The results of both conditions (i.e., with and without visual information) were statistically significant with respect to the original mix (VIR = 0 dB), as revealed by a two-tailed *t*-test (*p* < 0.005). When comparing the mean VIR between both conditions, however, no significant differences were found (*p* = 0.024).

## 4. Discussion

In this work, a real-time source separation algorithm based on a DNN has been designed to enhance the singing voice in pop western music for CI users. The real-time implementation allowed the investigation of remixing music for CI users under realistic acoustic environments and with the presence of additional visual information. Moreover, for the first time, the subjects were able to modify the amount of vocal enhancement online in contrast to previous studies that only used pre-processed sounds. The results of the current study confirm that CI users prefer the vocals enhanced with respect to the instruments even if the music contains reverberation and visual information is available.

The proposed algorithm to remix music for CI users is based on an MLP with an input layer with 513 × 3 units, one hidden layer and an output layer with dimension 1,026. Based on the proposed benchmark by Gajȩcki and Nogueira (2018), CI users should not be able to notice the degradation in sound quality caused by the source separation algorithm when the SDR and SAR are larger 0.69 and 4.42 dB, respectively. The proposed algorithm obtained an SDR of 8 dB using the iKala test data set and an SDR of 5.5 and 3 dB for the MUSDB and the Buyens data sets, respectively. Moreover, the source separation algorithm could be implemented in the front end of a sound coding strategy as its algorithmic latency is determined by the hop size of the used STFT. In our implementation, we used a hop size equal to 25% of the STFT's window length resulting in 6 ms algorithmic latency. The algorithm was implemented in MATLAB and run at 44,100 Hz sample rate in a 64 bit Windows 10 PC with an Intel (Santa Clara, California, USA) Core i7 4.3 GHz CPU and 16 GB RAM resulting in a computation time of 2 ms for each audio frame, well below the algorithmic latency to ensure real time processing. Hay-McCutcheon et al. (2009) measured and reported the minimum noticeable audiovisual asynchrony for CI users. The outcomes of that research revealed that CI users were insensitive to an asynchrony of up to 200 ms when the video was leading the audio. Considering that result and the latency caused by our system (around 100 ms), we assume that the audiovisual asynchrony of our system was not noticeable for the tested CI subjects. Moreover, it is worth mentioning that during listening experiments, none of the subjects expressed any reaction regarding the audiovisual asynchrony caused by the latency.

Ten bilateral CI users participated in two perceptual experiments. The first experiment showed that even if reverberation is added to the music scene, CI users prefer the vocals enhanced with respect to the background instruments. Two methods were used to alter the VIR, in the first method the instruments were kept constant while the singing voice was modified. Under this condition, CI users set the VIR to 8.2 and 8.5 dB for a low and a high reverberant room, respectively. Note that, as one of the subjects (S7) had bilateral residual hearing in the low frequencies, earplugs were used to attenuate the sounds transmitted through his/her acoustic hearing. Still, the attenuation was probably not enough to completely attenuate the low-frequency acoustic sounds causing the subject to set the VIR to high values to be able to perceive the voice enhancement more clearly.

In this same condition, NH listeners set the VIR to −2 and −1.7 dB for the low and the high reverberant room, respectively. The main limitation of this method to alter the VIR is that the presentation level increased with increasing VIR and therefore, presentation level was a confounding factor. For this reason, the experiment was repeated modifying the VIR by altering the singing voice and the background instruments level simultaneously but in opposite directions to keep the music presentation level constant. In this second condition, CI users preferred a VIR of 5.3 and 4.6 dB in the low and the high reverberant sound scenario, respectively. In contrast, NH listeners did not prefer the vocals to be enhanced. Previous studies (Buyens et al., 2014; Pons et al., 2016) showed that CI users find music more enjoyable when the vocals are enhanced by 6 dB on average. The larger vocal enhancement observed in the current study may be explained by the introduced reverberation which causes more difficulties in perceiving the singing voice and in turn results in CI users requiring an even further enhancement of the singing voice.

In the second experiment, the impact of visual information was examined by comparing the VIR preferences of CI users with and without using VR headset. The results of the experiment show no significant difference between the measured VIR with and without visual information. These results indicate that the use of source separation to remix music in CI listeners to enhance the singing voice may be applicable also for music listening in live concerts, performances, theaters, religious ceremonies or any other social event related to music that contains visual information.

It is important to mention that each subject had distinct VIR preferences and that the preferred VIR even varied from music track to music track. These results indicate that each CI recipient needs a subject-specific remixed music track for a better music appreciation. For this reason, it is important to expose the VIR parameter of the source separation algorithm such that the CI listener can adjust it to its own needs. Here one can foresee that the wireless communication to smartphones or the use of remote controls with such a parameter exposed could be very beneficial to make music more accessible for CI users (Nogueira et al., 2019).

## 5. Conclusion

This work introduced a real-time music source separation algorithm using a multilayer perceptron (MLP) to separate the singing voice from the background elements in music for CI users in realistic sound scenarios. Objective results show that the implemented neural network fulfills the benchmark reported by Gajȩcki and Nogueira (2018) and therefore, we assume that the degradation caused by this algorithm is not noticeable by CI recipients. Results from the experimental measures in CI users show that neither the presence of visual information nor different sound scenarios have an impact on VIR adjustment by CI recipients. Our experiments confirm that CI recipients find music more enjoyable when the vocals are enhanced with respect to the instruments and that this can be achieved by real-time audio source separation based on a neural network.

## Data Availability Statement

The raw data supporting the conclusions of this article will be made available by the authors, without undue reservation.

## Ethics Statement

The studies involving human participants were reviewed and approved by the Medical University Hannover Institutional Review Board. The patients/participants provided their written informed consent to participate in this study.

## Author Contributions

ST contributed with the study design, measurements, algorithm design, implementation, and writing the manuscript. TG contributed in the algorithm development, study design, and writing the manuscript. WN contributed with the project and study design, algorithm development, and writing the manuscript.

## Conflict of Interest

The authors declare that the research was conducted in the absence of any commercial or financial relationships that could be construed as a potential conflict of interest.
